# Comparative Study of the Impact of Human Leukocyte Antigens on Renal Transplant Survival in Andalusia and the United States

**DOI:** 10.3390/diagnostics13040608

**Published:** 2023-02-07

**Authors:** Alejandro Talaminos Barroso, Javier Reina Tosina, Laura M. Roa, Jorge Calvillo Arbizu, Miguel Angel Pérez Valdivia, Rafael Medina, Jose Luis Rocha Castilla, Pablo Castro-de-la-Nuez

**Affiliations:** 1 Biomedical Engineering Group, Department of Signal Theory and Communications, University of Seville, 41004 Seville, Spain; 2 Department of Signal Theory and Communications, University of Seville, 41004 Seville, Spain; 3 Department of Telematics Engineering, University of Seville, 41004 Seville, Spain; 4 Urology and Nephrology Department, University Hospital Virgen del Rocío, 41012 Seville, Spain; 5 Autonomous Regional Transplant Coordination in Andalusia, 41012 Seville, Spain

**Keywords:** renal transplantation, survival analysis, blood type, human leukocyte antigens, socio-economic factors

## Abstract

Renal transplantation is the treatment of choice for patients suffering from chronic renal disease, one of the leading causes of death worldwide. Among the biological barriers that may increase the risk of acute renal graft rejection is the presence of human leukocyte antigen (HLA) incompatibilities between donor and recipient. This work presents a comparative study of the influence of HLA incompatibilities on renal transplantation survival in the Andalusian (South of Spain) and United States (US) population. The main objective is to analyse the extent to which results about the influence of different factors on renal graft survival can be generalised to different populations. The Kaplan–Meier estimator and the Cox model have been used to identify and quantify the impact on the survival probability of HLA incompatibilities, both in isolation and in conjunction with other factors associated with the donor and recipient. According to the results obtained, HLA incompatibilities considered in isolation have negligible impact on renal survival in the Andalusian population and a moderate impact in the US population. Grouping by HLA score presents some similarities for both populations, while the sum of all HLA scores (aHLA) only has an impact on the US population. Finally, the graft survival probability of the two populations differs when aHLA is considered in conjunction with blood type. The results suggest that the disparities in the renal graft survival probability between the two populations under study are due not only to biological and transplantation-associated factors, but also to social–health factors and ethnic heterogeneity between populations.

## 1. Introduction

Transplantation is the most appropriate treatment for end-stage renal disease, one of the deadliest diseases in the world. The low availability of organs for transplantation has led to long waiting lists and transplant tourism in some countries. In Europe and the United States (US), legislative, regulatory and humanitarian services are provided to expedite the transplantation process and the appropriate choice between donor and recipient [1]. However, organ shortages, under-represented minority groups, difficulties in obtaining consent, lack of information, general ethical concerns, religion, problems of organ transportation and other socio-cultural and political factors have led to a lack of organ availability [2] and are counted among the main non-biological barriers that lengthen the waiting time before transplantation.

The process of admission, surgery, and post-transplant treatment differs between the US and most European countries. In 2019, 60% of transplant recipients in the US had public coverage under the federal Medicare program, while 32% were covered by private insurance [3]. Medicare coverage ends 3 years after transplantation, except for patients aged 65 or older, or for disability [4], making it difficult the access to expensive immunosuppressive drugs, which are key to long-term graft survival. In late 2020, US Congress passed the Comprehensive Immunosuppressive Drug Coverage for Renal Transplant Patients Act (Immuno Bill) [5] to provide lifetime coverage of immunosuppressive drugs for renal transplant recipients. In Europe, most countries offer almost universal publicly funded renal transplant coverage, with the exceptions of Germany and Liechtenstein, where part of the costs are covered by private insurance [6].

Once patients have been added to the waiting list, access to transplantation is determined by the renal allocation scheme. Countries such as the United Kingdom, France and Spain have developed their own national allocation algorithms, while other countries such as Austria, Belgium, Croatia, Germany, Hungary, Luxembourg, the Netherlands and Slovenia are involved in international organ exchange initiatives such as Eurotransplant and Scandiatransplant [7]. In Spain, the search for compatible deceased donors is carried out using predictive tools that analyse the compatibility between donor and recipient [8], reducing the availability of organs for some of the patients such as patients with blood group B and highly sensitized patients. Even so, the percentage of renal grafts discarded in Spain is close to 25% in the case of brain death (DBD) and controlled asystole donation, reaching 44% in the case of uncontrolled asystole donation, with significant variability between centres [8]. In line with other countries, some regions of Spain, such as Andalusia, have also initiated a national program for the distribution of donor grafts in DBD that prioritizes patients with a higher degree of immunization. The pooling of organs and the creation of centralised waiting lists optimise the human leukocyte antigen (HLA) match between donor and recipient, which is generally considered the gold standard for donor–recipient matching [9]. Additionally, another important barrier in transplantation is blood group compatibility between donor and recipient [10]. The incidence of blood groups and HLA types varies across different countries [11], reducing the availability of organs for recipients.

There are two HLA molecules [12]: class I and class II antigens. The HLA-A and HLA-B antigens, belonging to class I, and the HLA-DR antigen, belonging to class II, are generally considered for the study of renal survival. In particular, a higher degree of HLA mismatch is associated with a higher frequency of failure [9]. The values of these HLA incompatibilities (HLA-A, HLA-B and HLA-DR) are quantified as class 0, 1 and 2, where class 0 represents a high degree of compatibility between donor and recipient, while class 2 indicates a low degree. In Europe, weights are assigned to A, B, and DR incompatibilities, while in the US, DR incompatibility is primarily considered [9].

Studies of this nature have been characterised by the independent analysis of HLA incompatibility and blood group, and to the best of the authors’ knowledge, there is no study that combines them or that analyses the effects of the sum of HLA incompatibilities (aHLA) considering two very different population databases. In this sense, the aim of this work is to analyse the effect of HLA on the probability of renal survival in the American and Andalusian populations, while considering the different HLA-A, HLA-B and HLA-DR incompatibilities in isolation and, subsequently, jointly in different time horizons and together with other factors such as blood group. The work attempts to discuss some of the possible causes of the differences in behaviour between the two populations and the difficulties involved in the analysis of two such heterogeneous populations. In a previous paper [13], the authors presented a preliminary study limited to the Andalusian population, and now the study is extended to include two distinct populations.

The paper is structured as follows: the methodology followed in this study is briefly described in Section 2. Section 3 starts with a description of the available data and subsequently presents the results obtained from the Kaplan–Meier estimator and the Cox model, comparing the results with those obtained by other authors. Section 4 discusses the outcomes. Finally, the conclusions highlight the differences in the probability of renal survival between the populations studied and the need for exploratory algorithms that combine multiple factors.

## 2. Methods and Materials

### 2.1. Methodology

In order to carry out this study, the general methodology for survival analysis was followed:

1. Bibliographic search of survival analysis studies that consider blood group and HLA incompatibilities as factors that have an impact on renal transplant survival.
2. Pre-processing of the available databases by performing cleaning and quality improvement process, including standardisation of date-type variables, identification of null values, elimination of unimportant variables, renaming of some variables in order to better describe their content, standardisation of values using the scaling technique and conversion of categorical variables.
3. Analysis of renal graft survival using the Kaplan–Meier estimator considering the different HLA incompatibilities, both in isolation and together as well as in combination with the blood group.
4. Obtaining the coefficients of the Cox model for each of the identified factors with greater significance identified in the previous step.
5. Graphical representation of the survival probabilities obtained and discussion of the results.

### 2.2. Data Characteristics

The renal transplant data that have been analysed in this work come from the Autonomous Transplant Database of Andalusia (SICATA), while the US data have been provided by the United Network for Organ Sharing (UNOS). For both databases, single renal transplants from cadaveric donors between 2006 and 2019 (both inclusive) were selected. The Regional Transplant Coordination Agency in Andalusia started database compilation for renal transplantation in 2006. No data are available for previous years. On the other hand, there has been a continued improvement in long-term success of renal transplantation in the last 10–15 years. Therefore, the situation before 2006 is not significant in light of today’s procedures. Likewise, both databases have been designed to cover the same time interval in order to ensure the greatest possible homogenization between them. Transplants from later dates have been discarded to avoid the possible influence of the COVID-19 pandemic on graft survival probability.

The number of transplants for SICATA is 3478, while for UNOS, there are 112,877 transplants. The number of transplant centres for SICATA is 6, while for UNOS, it is 270. The centres with the highest number of patients in SICATA are Virgen del Rocío Hospital (Seville) with 974 transplants, Puerta del Mar Hospital (Cadiz) with 765 transplants and Virgen de las Nieves Hospital (Granada) with 676 transplants. In UNOS, the names of the centres are encrypted. The centre with the highest number of operations reaches 2208 transplants. On the other hand, the average number of days on the waiting list for SICATA is 2.6 years, while for UNOS, it is 3.6 years [14].

The data refer to single renal transplants from donors older than 3 years and recipients older than 18 years, considering only donors and recipients with the same blood type. Table 1 presents the main characteristics for both databases, showing the homogeneity between percentages except in the proportion between donors and recipients of groups A and O. These values are the average of the two databases, not allowing for statistical analysis of them.

In SICATA, there is an equal distribution between these two groups, both for donors and recipients, whereas in UNOS, there is a predominance of group 0. This characteristic does not correspond to the distribution of blood groups in the Spanish and American populations, which have a similar distribution. Another notable dissimilarity between the two populations is in the age of donor, where there is a difference of approximately 14 years.

HLA typing from the SICATA database is based on DNA-based methods, specifically RFLP-PCR (restriction fragment length polymorphism polymerase chain reaction) and matching at the antigen level. The serological tests for HLA typing and anti-HLA antibodies are performed before transplantation. Finally, the Regional Transplant Coordination Agency notes that there is excellent monitoring of vital status and type of renal replacement therapy, but they compile no data about post-transplant complications. With regard to the UNOS database, in 2011, the OPTN mandated DNA-based HLA typing methods for deceased donors [15]. Typical methods used for HLA typing of deceased donors in the US include reverse sequence-specific oligonucleotide (rSSO) probe hybridization, sequence-specific primer (SSP)-directed PCR amplification, and SSP-directed real-time PCR (RT-PCR) amplification [16]. Due to its simplicity and speed, the RT-PCR method is used as the general approach for HLA typing of deceased donors in most US histocompatibility laboratories [16].

## 3. Results

### Results of the Data Analysis

**Figure 1:**
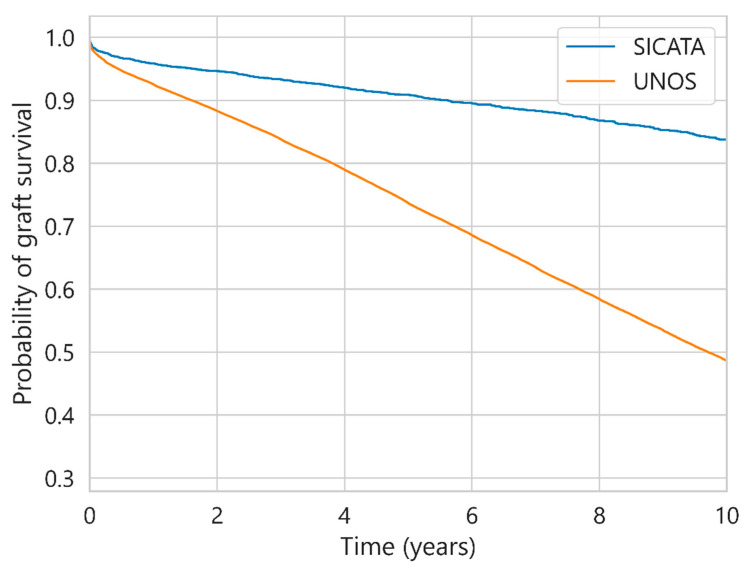
Probability of graft survival for UNOS and SICATA.

Figure 1 shows the survival probabilities for UNOS and SICATA for all data (hereafter referred to as the base curves). The probability curves diverge almost from the first day, with the differences increasing progressively over the entire period.

**Figure 2:**
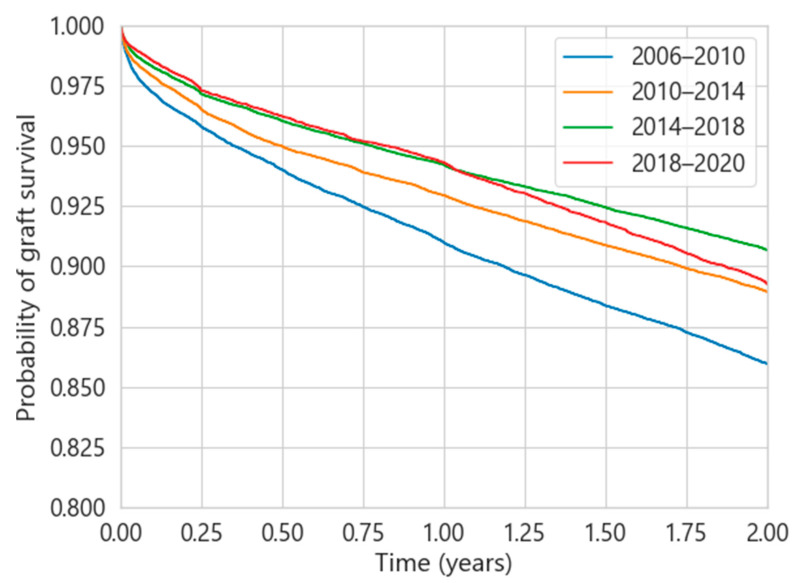
Probability of graft survival in the first two years for UNOS at different time intervals.

Figure 2 presents the evolution of the two-year renal survival probability at different time intervals for the UNOS database. The progressive improvement in the probability of graft survival is evident since 2006, reaching the highest differences between the period 2006–2010 and 2014–2018. It is expected that this trend will continue in the following years due to the Immuno Bill, mentioned in Section 1. These differences in behaviour between years are not observed for SICATA for the same time intervals.

**Figure 3:**
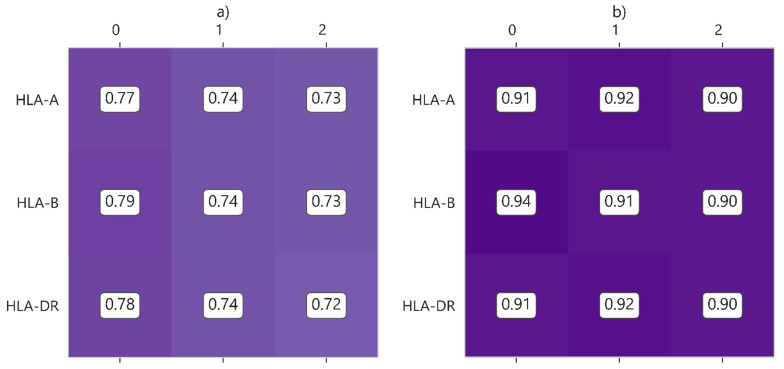
Five-year survival probability considering different values of HLA incompatibilities, for (**a**) UNOS and (**b**) SICATA.

**Figure 4:**
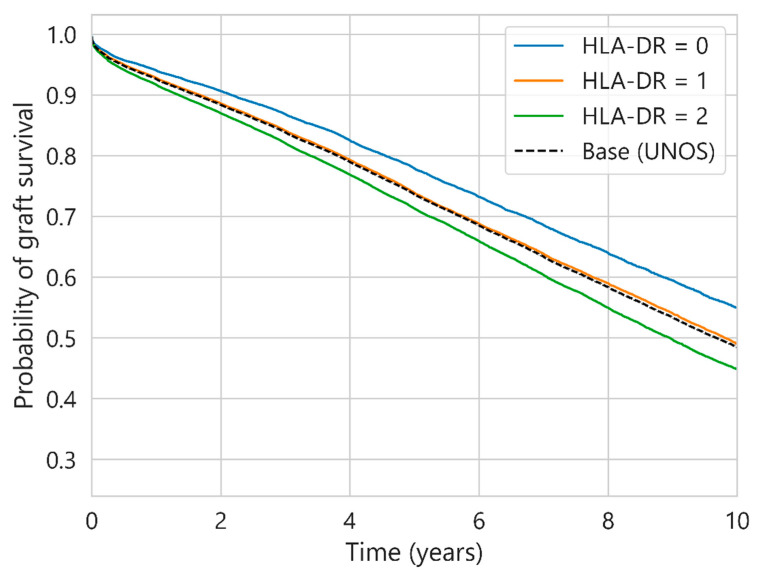
Ten-year survival probability for UNOS considering different HLA-DR mismatch values.

With respect to the levels of HLA incompatibilities (HLA-A, HLA-B and HLA-DR), the differences in the probability of survival for UNOS and SICATA are not very relevant considering the different values in isolation. Figure 3 shows the five-year survival probability values for UNOS (a) and SICATA (b), and the evolution of the 10-year survival probability for this incompatibility in UNOS is presented in Figure 4.

**Figure 5:**
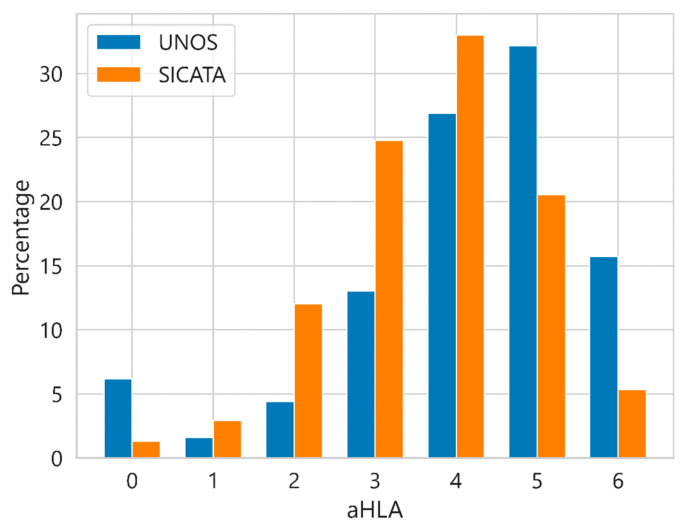
Percentage of cases with respect to the total considering the different aHLA values in UNOS and SICATA.

Survival analysis for aHLA (HLA-A + HLA-B + HLA-DR), with values between 0 and 6, which offers differences in behaviour as pointed out by some authors [17,18]. Figure 5 shows the distribution of cases by percentage considering the different aHLA values in UNOS and SICATA. The most notable differences between UNOS and SICATA are seen at all levels, where UNOS reaches a higher percentage in incompatibilities 0 and 6 compared to SICATA, while in SICATA, the highest percentages compared to UNOS are in the intermediate values.

**Figure 6:**
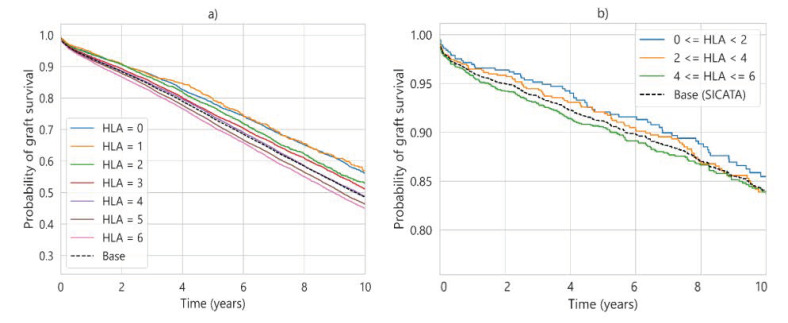
Ten-year survival probability considering different aHLA values, where (**a**) UNOS (*p* < 0.005) and (**b**) SICATA (*p* > 0.1).

The 10-year survival probability for UNOS (a) and SICATA (b) considering aHLA is presented in Figure 6. Given the scarcity of data for SICATA, three groupings have been chosen instead of six, while in UNOS, one grouping is maintained for each aHLA value.

The renal survival probability at one, three and five years considering the different aHLA combinations for UNOS and SICATA is shown in Table 2 and Table 3, respectively. The downward trend in the probability of survival in UNOS is notable for the different levels of aHLA in all years, with the differences at the extreme levels (one and six) becoming more pronounced as the years progress. Individual analysis of the different HLA combinations reveal that transplants with zero HLA-DR mismatches had significantly better survival than those with one or two, with independence of the values of HLA-A and HLA-B mismatches.

**Figure 7:**
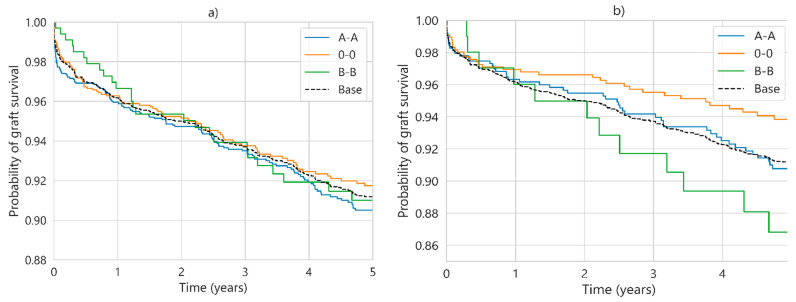
Five-year survival probability considering by blood group for SICATA, where (**a**) captures all cases (*p* > 0.5), and (**b**) shows only cases where aHLA is three or lower (*p* < 0.05).

Combining multiple factors to make population groupings can reveal significant differences that are not obtained by individual factor analysis. In SICATA, blood type has virtually no relevance for survival probability (Figure 7a), nor does aHLA (Figure 6b). However, the combination of both factors reveals changes in behaviour (Figure 7b), with a difference of 7% between the highest (blood group O) and the lowest (blood group B) survival population group, while blood group A transplants have a virtually overlapping survival probability compared to the base curve (all cases). These results have been presented by the authors of the present work in a previous paper [13]. No such differences were observed in UNOS.

Table 4 presents the hazard ratios of the most studied factors in the literature extracted from the Cox model considering three-year censored renal graft survival. In SICATA, the most significant variables are the age of the donor and recipient, as reported by other authors for the same data [19], while in UNOS, in addition to the two previous variables, the sex of the donor and recipient, the BMI of the recipient and HLA-DR incompatibility are also relevant. This multivariate Cox regression analysis shows that HLA-DR incompatibility has the most significant impact on renal survival with respect to HLA-A and HLA-B, as demonstrated by the higher hazard ratio value (1.12 vs. 1.02).

**Figure 8:**
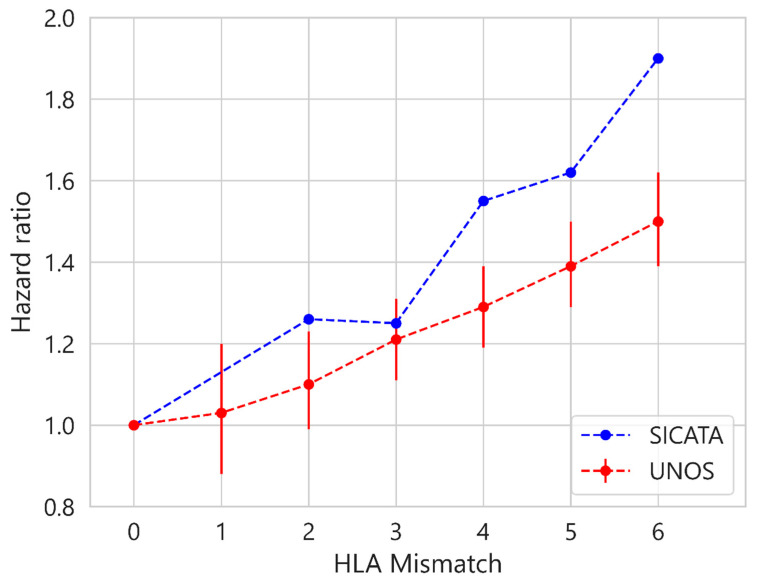
Hazard ratio for SICATA and UNOS at different levels of HLA incompatibilities.

Finally, Figure 8 shows the hazard ratio obtained from a univariate Cox model for each of the aHLA levels, taking 0 as the reference value. For SICATA, the value 1 has been omitted due to the scarcity of data for this level. The linear relationship of hazard ratios in UNOS as aHLA increases was obtained by other authors [20], although with older data (up to 2013). This linear trend is also observed for SICATA, with the hazard ratio being higher than in UNOS at all levels, although statistically insignificant (*p* > 0.05). The error bars are the 95% confidence intervals for each value of the hazard ratio, omitted for SICATA given the magnitude of these.

## 4. Discussion

Logistic and structural factors between the two populations can impact the survival probability curve, including the omission of HLA-B in the US allocation and the changes derived from the introduction in 2014 of the new kidney allocation system (KAS) in the US [21], which allows for improved long-term graft survival by matching better kidneys with less sick patients and by giving priority to national allocation for highly sensitized patients. Other possibly related factors are the cold ischemia time differences and the distances between clinical centres in the US and Spain, which would increase the time before transplantation. On the other hand, complex causes can include not only biological, but also socio-cultural, political, nutritional and religious factors. All these differences are remarkable in terms of the evolution of the probability of survival in recent years for the two populations studied.

In the US, the improvement in survival probability (Figure 2) has been progressive in recent years, reaching rates close to 90% for transplants after 2012 during the first two years post-transplantation [22]. The observed improvement is attributed to decreased clinical acute rejection rates, improved pre-transplant cross-matching techniques, surveillance for viral infections and effective antiviral prophylaxis [23,24]. On the other hand, in the Andalusian population, there has been practically no improvement in recent years. This behaviour may be due to the development of proactive initiatives associated with the transplant process in Spain through educational and training activities [25], with active support from public institutions. This has generated an impression of trust, transparency and altruism in society, which has made Spain the world leader in the number of organ donations per million inhabitants [26]. Other factors that have been identified as successful in Spain have been the broadening of the criteria for organ donation, the creation of protocols to promote organ donation after circulatory death, and the identification of potential organ donors outside the intensive care unit setting [27]. These divergent results in the two populations reveal that some regions have already reached a high degree of maturity in the full renal transplantation process, adjusting the action protocols to the social, cultural and health conditions of the territory. The margin for improvement that exists in other parts of the world, and which is evident in the continuous improvement in the probability of survival in recent years, could be partially addressed by analysing the experience of other regions where a stable probability of survival has already been achieved.

With respect to the levels of HLA incompatibilities, the most notable differences between values are reached in the HLA-DR mismatch for UNOS. HLA-DR mismatches in particular were significantly associated with worse renal graft survival in the US [28]. The trends in UNOS are in agreement with the results obtained from others authors [29]. The impact on renal survival has still been maintained since the last two decades [30], except between 1994 and 1998 [31]. The reasons for this individual impact of HLA-DR with respect to the other two types of mismatches are under study [32], being also common in transplants of other organs. Some authors [33] postulate that DR antigens may be more immunogenic than A or B antigens. This work also suggests a strong imbalance in the HLA complex and the existence of a confounding relationship in which a mismatch in HLA-DR might increase the probability of a mismatch in HLA-DQ, a locus that is not so well studied in the literature and is not included in the donor–recipient assignment. This relationship between HLA-DR and HLA-DQ would amplify the effects on renal graft survival.

In the aHLA survival analysis, it is well established in the literature that, in deceased donor renal transplants, zero or one aHLA mismatch offers a significant benefit in graft survival with respect to transplants with a higher number of mismatches [34]. However, recent advances in the field of immunosuppression appear to be diminishing this influence, and some studies of renal transplants from living donors reported no influence of aHLA on renal survival [35]. On the other hand, to the best of the authors’ knowledge, there are no similar studies for the Andalusian population, but there are studies that analyse the impact of HLA incompatibilities on renal transplants in Spain [20,36], and no significant influence was found.

These behavioural differences in the impact of aHLA on renal survival between UNOS and SICATA may be due to ethnical factors. SICATA does not collect data in this regard, as it is understood that this factor is not very significant given the majority of Caucasians in Spain. In the UNOS database, this factor is included for both donor and recipient, with a predominance of Caucasians, although with greater ethnic heterogeneity than in Spain. Some studies conclude that the best HLA matches occur when the donor and recipient are of the same race when the transplant comes from a cadaveric donor [37]. In addition, a recent study [38] indicated that HLA-based allocation systems disadvantage Afro-American patients, and this trend has been ongoing for decades [37]. The study reveals that HLA matching is more difficult for Afro-American patients than for white patients, with a lower likelihood that an Afro-American recipient will receive a transplant from a donor with a zero HLA mismatch.

## 5. Conclusions

This paper has presented the analysis of the influence of HLA incompatibilities on renal graft survival in the American population (UNOS database) and in the Spanish region of Andalusia (SICATA database). The study included a Kaplan–Meier analysis for each incompatibility in isolation (HLA-A, HLA-B and HLA-DR), together, with combinations of values and in combination with the blood group. A study based on the Cox regression method quantified the influence of the different HLA incompatibilities in relation to other factors that are generally included in other studies.

The results indicate that each increase in HLA incompatibility was significantly associated with a higher probability of graft failure in UNOS, while no relevant differences were observed in SICATA. These behaviours may be due to the ethnic diversity between the two populations that have been analysed. Cox regression modelling revealed that HLA-DR incompatibility was the most important factor in renal graft survival for UNOS (*p* < 0.005), while for SICATA, it was HLA-B, although not significantly (*p* = 0.1).

The work shows that the discrepancies between the different databases are not only due to biological factors. In this sense, the conclusions obtained for each database in isolation cannot be generalized to the other due to cultural, religious, socio-political, ethnic and/or nutritional differences between the two populations that have been the subject of this study. Other clinical factors that could also be have an effect include the time on dialysis prior to transplant, the proportion of high immunological risk transplants considering highly sensitized patients undergoing retransplantation, and cold ischemia time, which should be significantly longer in the US than in Spain due to the long distance travelled for organs. In addition, the high statistical significance in SICATA (*p* < 0.005) taking aHLA scores of three or lower in combination with blood group revealed that the analysis of some factors has no relevance for renal survival when analysed in isolation but may have significance when combined with other factors. The work highlights the need for exploratory algorithms that combine multiple factors to find possible behaviours of interest that are hidden with an individual analysis. The creation of a multi-parametric algorithm could offer a better hypothetical prognosis for renal transplantation.

## Figures and Tables

**Table 1:** Donor and recipient characteristics.

Characteristic	UNOS (112,877 Cases)	SICATA (3478 Cases)
Age of donor	38.93 (±15.42)	53.32 (±15.02)
Gender of donor (% men)	68,903 (61.04%)	2143 (61.62%)
BMI of donor (kg/m^2^)	27.77 (±6.82)	27.4 (±15.02)
Age of recipient	52.81 (±13.14)	52.74 (±12.89)
Gender of recipient (% men)	68,289 (60.5%)	2205 (63.4%)
BMI of recipient (kg/m^2^)	28.11 (±5.4)	27.18 (±7.87)
Blood group A–A	20,461 (18.13%)	1624 (46.69%)
Blood group B–B	18,570 (16.45%)	335 (9.63%)
Blood group O–O	71,386 (63.24%)	1386 (39.85%)
Blood group AB–AB	2460 (2.18%)	133 (3.82%)
HLA-A: class 0	14,966 (13.26%)	568 (16.33%)
HLA-A: class 1	42,965 (38.06%)	1760 (50.6%)
HLA-A: class 2	54,946 (48.68%)	1150 (33.06%)
HLA-B: class 0	10,216 (9.05%)	247 (7.1%)
HLA-B: class 1	27,492 (26.36%)	1140 (40.54%)
HLA-B: class 2	75,169 (66.59%)	1821 (52.36%)
HLA-DR: class 0	19,658 (17.42%)	647 (18.6%)
HLA-DR: class 1	51,656 (45.76%)	1965 (56.5%)
HLA-DR: class 2	41,563 (36.82%)	866 (24.9%)

**Table 2:** Censored survival probability to renal graft failure at one, three and five years for UNOS from aHLA.

aHLA	Cases	Probability of Survival		
		1 Year	3 Years	5 Years
1	1789	0.95	0.88	0.81
2	4927	0.94	0.87	0.78
3	14,627	0.94	0.85	0.75
4	16,236	0.93	0.85	0.74
5	36,085	0.93	0.84	0.73
6	17,644	0.92	0.83	0.72

**Table 3:** Censored survival probability to renal graft failure at one, three and five years for SICATA from aHLA.

aHLA	Cases	Probability of Survival		
		1 Year	3 Years	5 Years
1	102	0.99	0.98	0.98
2	419	0.94	0.93	0.91
3	862	0.96	0.95	0.92
4	617	0.97	0.91	0.89
5	715	0.95	0.92	0.89
6	186	0.94	0.92	0.9

**Table 4:** Cox multivariate proportional hazards analysis of censored graft survival to failure for SICATA and UNOS at three years for all data.

Factor	SICATA		UNOS	
	Hazard Ratio	*p*	Hazard Ratio	*p*
Age of donor	1.04 (1.02–1.05)	<0.005	1.01 (1.01–1.01)	<0.005
Gender of donor	0.96 (0.69–1.34)	0.81	1.11 (1.07–1.14)	<0.005
BMI of donor	1.01 (0.97–1.04)	0.69	1 (1–1)	0.75
Age of recipient	0.98 (0.96–0.99)	<0.005	1.01 (1.01–1.01)	<0.005
Gender of recipient	1.47 (1.06–2.02)	0.02	0.88 (0.85–0.91)	<0.005
BMI del receptor	1 (0.99–1.02)	0.76	1.01 (1.01–1.01)	<0.005
HLA-A	1.1 (0.87–1.39)	0.44	1.02 (1–1.05)	0.09
HLA-B	1.26 (0.93–1.56)	0.1	1.02 (0.99–1.05)	0.2
HLA-DR	1.2 (0.93–1.54)	0.16	1.12 (1.1–1.15)	<0.005

## Data Availability

The data used for the current study can be made available by the corresponding author on reasonable request.
